# Optimization of Ultrasound Extraction of Total Anthocyanin From *Berberis kaschgarica* Rupr. by Response Surface Methodology and Its Antihypertensive Effect

**DOI:** 10.1002/fsn3.4591

**Published:** 2024-11-22

**Authors:** Nawaz Khan, Maierdan Yusufu, Zeynab Ahmadova, Nulibiya Maihemuti, Sendaer Hailati, Ziruo Talihat, Nuerbiye Nueraihemaiti, Dilihuma Dilimulati, Alhar Baishan, Li Duan, Wenting Zhou

**Affiliations:** ^1^ Department of Pharmacology, School of Pharmacy Xinjiang Medical University Urumqi Xinjiang China; ^2^ Department of Orthopedics, Guangdong Artificial Intelligence Biomedical Innovation Platform, Shenzhen Second People's Hospital The First Affiliated Hospital of Shenzhen University Shenzhen Guangdong China; ^3^ Medical Innovation Technology Transformation Center of Shenzhen Second People Hospital Shenzhen Shenzhen Guangdong China; ^4^ School of Medicine Shenzhen University Shenzhen Guangdong China; ^5^ Center for Disease Control and Prevention of Midong District Urumqi Xinjiang China; ^6^ Xinjiang Key Laboratory of Natural Medicines Active Components and Drug Release Technology Urumqi Xinjiang China; ^7^ Xinjiang Key Laboratory of Biopharmaceuticals and Medical Devices Urumqi Xinjiang China; ^8^ Engineering Research Center of Xinjiang and Central Asian Medicine Resources Ministry of Education Urumqi Xinjiang China

**Keywords:** anthocyanin, *Berberis kaschgarica* Rupr., environmental sustainability, hypertension, ultrasound‐assisted extraction

## Abstract

*Berberis kaschgarica* Rupr. is a berry fruit shrub found in the north‐western region of China, locally its fruit is consumed as a tea ingredient a part of the daily diet, for treatment of different diseases like eczema, and for cardiovascular care as a traditional remedy. In the current study, an optimized ultrasound‐assisted extraction (UAE) method is developed using response surface methodology (RSM) to extract anthocyanins from the fruit. The influence of the extraction conditions on the yield of anthocyanins was evaluated and discussed. Additionally, the antihypertensive activity of the extract was evaluated in rats. The negative control group comprised Wistar–Kyoto rats, while five experimental groups included spontaneously hypertensive rats: model group; captopril group; and three treatment groups receiving purified extract. Blood pressure was regularly monitored every 2 weeks during the 12‐week treatment period. Post‐treatment left ventricular cardiac function in rats was assessed, and serum level of renin (REN), angiotensin‐converting enzyme (ACE), angiotensin II (Ang‐II), angiotensin‐(1–7) (Ang‐(1–7)), endothelin‐1 (ET‐1) and nitric oxide (NO) were evaluated. The optimal UAE extraction condition determined by RSM was 56.5% ethanol volume, 1:19.74 solid‐to‐liquid ratio, 55 min extraction time, and 0.71% of HCL volume. The purified *B. kaschgarica* anthocyanin extract exhibited antihypertensive effects and significantly improved left ventricular cardiac functions in treated rats. Furthermore, serum levels of REN, ACE, Ang‐II, ET‐1, Ang‐(1–7), and NO showed statistically significant changes in the treated groups. This study highlights the efficacy of the developed optimized UAE method for high‐yield anthocyanin extraction from *B. kaschgarica* fruit and its potential as a therapeutic agent for hypertension.

## Graphical abstract

In the current research, an optimized ultrasound‐assisted extraction method was developed for extracting anthocyanin from *Berberis kaschgarica* Rupr. fruits. The extract was purified and its effects were evaluated against hypertension in the rat model. The developed UAE method was found suitable for high‐yield anthocyanin extraction. The extract was found as a major source of anthocyanin with impressive antihypertensive effects.

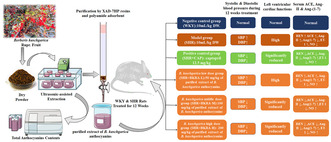

## 1 Introduction

Green extraction methods represent a modern approach to the extraction process in the extraction of bioactive compounds from plant materials aiming to minimize energy consumption utilize alternative environmentally friendly solvents and produce renewable natural products while ensuring high quality and safe extracts (More, Jambrak, and Arya 2022). This innovation concept addresses the limitations of conventional extraction techniques by reducing power consumption, minimizing organic solvent usage, and eliminating harm to the environment (Jha and Sit 2022). Over recent years there has been a notable shift towards innovative and sustainable approaches in industries including food, pharmaceuticals, and cosmetics driven by the principle of green chemistry (Chemat, Vian, and Cravotto 2012). These practices focus on economic viability, environmental sustainability, and safety which led to the coining of the term “green extraction” and six established guiding principles for it (Nutrizio et al. 2021). These principles of green extraction involve the utilization of renewal and sustainable bioresources, the adoption of green solvents or water, reduced energy inputs, the production of coproducts from waste, the minimization of unit operations, and the production of nondenatured and biodegradable extracts (More, Jambrak, and Arya 2022; Chemat et al. 2019). Given the importance of these principles, researchers and scientists are intensifying their focus on optimizing extraction processes to enhance the efficacy and extract quality from plant resources. One recent technology demonstrating reduced solvent and energy usage is ultrasound‐assisted extraction (UAE) (More and Arya 2021). Ultrasound operates at a frequency above 20 KHz that induces cavitation bubbles which facilitate the release of bioactive compounds from the cells of plant materials by disrupting cell wall and membranes (Chemat et al. 2017b; Peng et al. 2023). This method has gained huge interest due to its effectiveness in the extraction of valuable bioactive compounds and minimizing environmental impacts (Shen et al. 2023). However, the yield of extraction of the bioactive compounds can be affected by various factors such as pH of the solvent, ratio of solvent and sample, temperature, or extraction time (Casagrande et al. 2018). Regardless of extensive research progress, there is no universally standardized set of optimum conditions for bioactive compound extraction from different plants that ultimately results in the necessitation of a species‐specific optimization method. Single factor at a time approaches are labor intensive and time consuming as well as lacking interactive effects (Pandey et al. 2018). Statistical models such as response surface methodology (RSM) offer an effective tool for optimizing complex processes by evaluating multiple parameters and their interactions in a single experiment (Mudaser et al. 2021). The method is widely used for optimizing the extraction of various compounds from different plant materials (Cannas et al. 2023). RSM enables efficient experimentation and interpretation with the Box–Behnken design (BBD) being particularly favored for its simplicity and ease of use.

The healthy food industry is experiencing rapid growth, especially in the domain of herbal food products. This trend is propelled by the abundance of bioactive components, including phenolic compounds, flavonoids, and anthocyanins known for their health‐promoting effects. These compounds contribute to the cure of different chronic diseases such as cardiovascular diseases, cancer, and other complications (Insang et al. 2022). Recently the use of medicinal agents derived from natural sources has been increased as an alternative to synthetic drugs. These naturally produced compounds are usually genetically encoded and are plant secondary metabolites, which mostly serve as primary sources for developing new drugs (Piątczak et al. 2020; Pye et al. 2017). Plant‐based secondary metabolites are classified into various groups based on their chemical compositions, that is, alkaloids, terpenoids, phenolics, glycosides, anthocyanins, and so on (Khan et al. 2022). Among these, anthocyanins have been noticed with beneficial health‐promoting activities. In plants, anthocyanins are mainly responsible for the red‐blue or orange coloration of seeds, fruits, flowers, or vegetables (Liu et al. 2018). Chemically, anthocyanins comprise anthocyanin glycosides, which are glycosylated structures, and anthocyanidins which are sugar‐free anthocyanidine aglycons and are non‐glycosylated structures (Liu et al. 2018; Oliveira et al. 2020). Major sources of anthocyanins include berry fruits such as blueberries, strawberries, and red grapes along with dates and nuts which are recognized as high‐concentration sources (Mozos et al. 2021). Moreover, it may also be used as a natural food colorant (Awika and Duodu 2017). However, the stability of anthocyanins is poor and is sensitive to various factors such as heat, light, pH, and so on, which brings great challenges to the extraction, preservation, and application of anthocyanins. Numerous studies both in vitro and in vivo support its health‐promoting effects against cardiovascular diseases (Wallace, Slavin, and Frankenfeld 2016), with evidence from epidemiological studies supporting their preventive effects on the onset of cardiovascular diseases in dose‐dependent manner both in men and women (Jennings et al. 2012; Mccullough et al. 2012; Mink et al. 2007; Cassidy et al. 2011).

**FIGURE 1:**
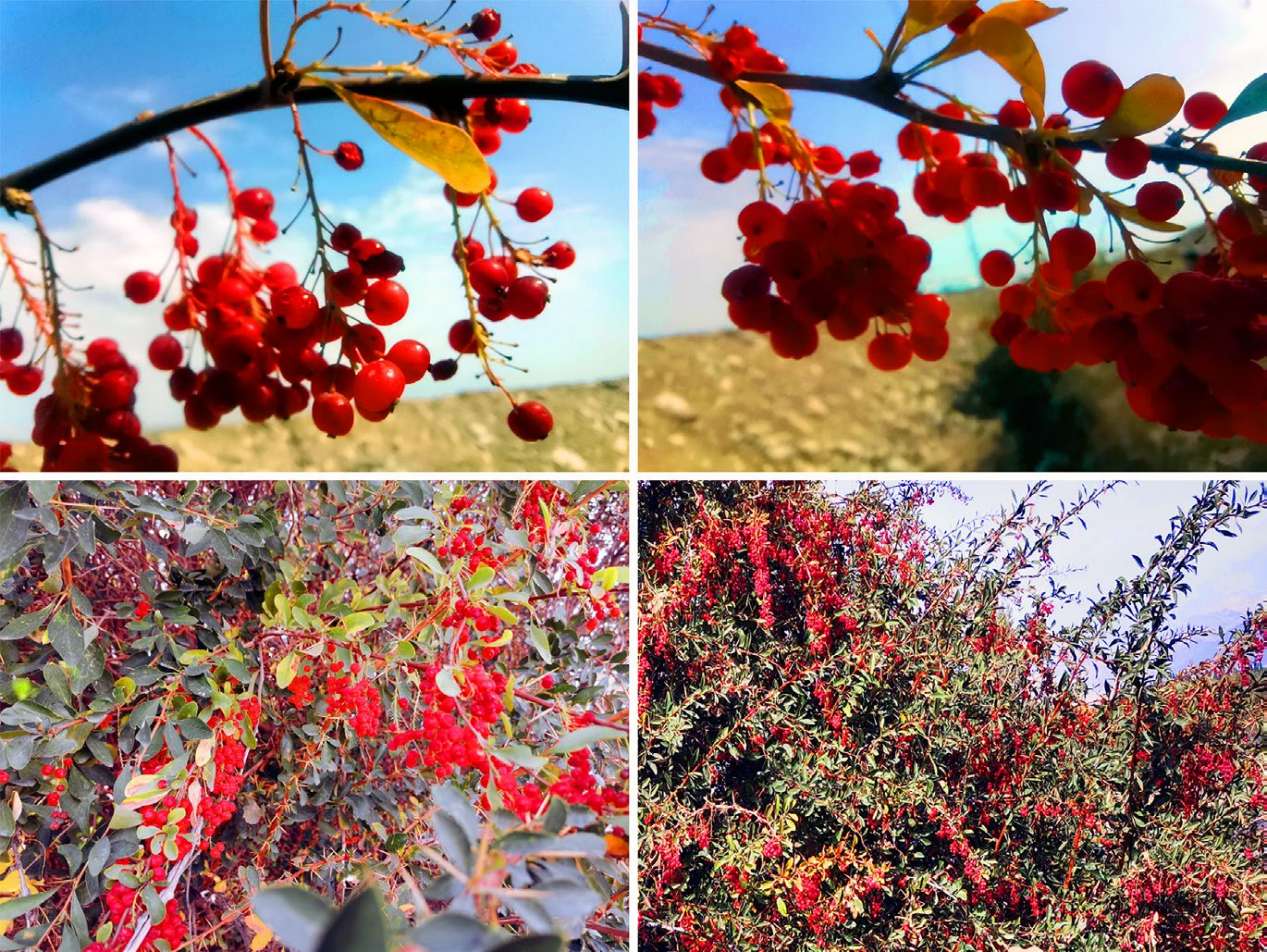
*Berberis kaschgarica* Rupr. plant and fruits.

*Berberis kaschgarica* Rupr. is a berry fruit shrub belonging to *Berberidaceae* family habitual to the north‐western region of China (Hou and Xue‐Yu 1983; Figure 1). In the region, the fruit of the plant is consumed as a tea ingredient, part of daily diet as well as utilized in the treatment of different diseases as a traditional remedy. It is commonly used in the treatment of skin conditions like eczema and for cardiovascular disease care, particularly hypertension. In the current study, we first aimed at the extraction of anthocyanins from *B. kaschgarica* fruit by developing an optimized UAE model. The parameters of UAE including the concentration of ethanol, HCL volume, extraction time, and solid‐to‐liquid ratio were optimized by RSM and single factor experiment. Subsequently, the extract underwent a purification process to obtain a high yield of anthocyanins. The purified extract of *B. kaschgarica* anthocyanins was investigated for its antihypertensive effect in spontaneously hypertensive (SHR) rats' model. To the best of our knowledge, this study marks the first investigation into the pharmacological activities of *B. kaschgarica*, particularly its antihypertensive effect.

## 2 Materials and Methods

### 2.1 Fruit Materials

The fruit of *B. kaschgarica* was sourced from the Kashghar county of Xinjiang, China and authenticated by Prof. Palida Abulizi, from the Department of Natural Medicinal Chemistry and Pharmacognosy, School of Pharmacy, Xinjiang Medical University. A Voucher specimen (NO: XJMUYXY20201020) was deposited in the Department of Traditional Chinese Medicines Ethnical Herbs Specimen Museum of Xinjiang Medical University. Subsequently, the fruits were shade dried at room temperature, ground to powder, and sieved in a 20‐mesh sieve before storage in a cool and dark place until use.

### 2.2 Extraction of Anthocyanins for *B. kaschgarica* Fruits by Ultrasound‐Assisted Extraction

Anthocyanin was extracted from the dried fruit powder using UAE, and followed a standardized protocol (Zhu et al. 2016). The extraction was performed using an ultrasonic device (KJ1004B, Kejin Instrument Company, China) with adjustable power (100‐500 W) and a frequency of 20 kHz.

Briefly, 10 g of the dried fruit powder was accurately weighed and dispersed in an acidified aqueous‐ethanolic solution (20%–80%) at a solid‐to‐liquid ratio ranging from 1:10 to 1:40 in a glass conical flask. The ultrasonic extraction was conducted for 10–60 min at a constant temperature (30°C–60°C). After cooling to room temperature, the extract was centrifuged for 10 min at 3000 rpm and filtered using Whatman No. 1 filter paper. The resulting samples were then used for the determination of total anthocyanin content.

### 2.3 Single Factor Experimental Design

To assess the impact of individual extraction parameters of UAE on anthocyanin yield, single‐factor experiments were conducted following a previous study (Zou et al. 2011). These parameters included ethanolic concentration, solid‐to‐liquid ratio, HCL volume, extraction temperature, ultrasonic power, and extraction time. Each experiment involved altering one specific parameter while maintaining the other constant as described in Section 2.2. Each experiment was conducted in triplicate.

### 2.4 Determination of Total Anthocyanin Contents of *B. kaschgarica* Fruit Extract

Total anthocyanin content was determined spectrophotometrically using the pH differential method with some minor modifications (Anuar et al. 2013). Briefly, 2 mL of the collected supernatant samples were individually diluted with 0.4 M of sodium acetate buffer (pH = 4.5) and 0.025 M potassium chloride buffer (pH = 1). After 15 min incubation period at room temperature in the dark, the absorbance was measured by UV–visible spectrophotometer at wavelengths of 500 and 700 nm.

The absorbance was calculated as 𝐴 = [(𝐴_500_ − 𝐴_700_) pH 1.0 − (𝐴_500_ − 𝐴_700_) pH 4.5]. The total anthocyanin content was determined using the following formula, with cyanidin‐3‐glucoside used as standard.

$$\text{Anthocyanin content}\;\left(\mathrm{mg}/g\right)=\text{34𝐴}\times \mathrm{MW}\times \mathrm{DF}\times \text{34𝑉 /34𝑉𝜀×34𝑉𝜀𝑙×34𝑉𝜀𝑙𝑚 sample}$$

where 𝐴 is the absorbance from the spectrophotometric measurement, MW is the molecular weight (449.2 g/mol of cyanidin‐3‐glucoside), DF is the dilution factor, 𝑉 is the extract volume (mL) that was brought as sample stock solution, 𝜀 is the molar absorptivity (26,900), 𝑙 is the cell path length (1 cm), and 𝑚 is the *B. kaschgarica* dried fruit powder weight (g).

### 2.5 Optimization of UAE by Response Surface Methodology

In the experiment, for optimizing the extraction parameters, RSM model was employed according to a standard protocol (Said and Amin 2015). A four‐factor with three‐level BBD was utilized. Ethanol concentration (*X*_1_), solid‐to‐liquid ratio (*X*_2_), extraction time (*X*_3_), and HCL volume (*X*_4_) were selected as independent variables. The independent variable ranges are shown in Table 1 and are as follows: ethanol concentration ranged from 50% to 70% by volume, solid‐to‐liquid ratio ranged from 1:15 to 1:25 g/mL, 45–55 min extraction time, and HCL volume ranged from 0.6% to 0.8% by volume. The center point values and the ranges displayed in Table 1 for the four independent variables were established on the basis of the preliminary single‐factor experiment results. The designed experiment consists of a total of 29 experiments with 4 central point replicants. To mitigate the effects of unanticipated variability in the observed responses, the experimental runs were randomized. The given equation was employed to code the variables:

$$X=\left(X_{i}-X_{o}\right)/∆X$$

**TABLE 1:** Actual and coded level of the four variables.

Variables	Factors	Coded levels		
		−1	0	1
Ethanol concentration (%)	*X*_1_	50	60	70
Solid‐to‐liquid ratio (g/mL)	*X*_2_	1:15	1:20	1:25
Extraction time (min)	*X*_3_	45	50	55
HCL volume (%)	*X*_4_	0.6	0.7	0.8

X is the coded value, $X_{i}$ is the corresponding actual value, $X_{o}$ is the actual value in the center of the domain, and ∆X is the increment of $X_{i}$ corresponding to a variation of 1 unit of X.

The corresponding mathematical model for Box–Behnken design is

$$Y=b_{o}\sum_{i=1}^{4}b_{i}X_{i}+\sum_{i=1}^{4}b_{\mathrm{ii}}X_{i}^{2}+\sum_{i=1}^{3}\;\sum_{m=i+1}^{4}b_{\mathrm{im}}X_{i}X_{m}$$

*Y* (yield) represents the dependent variable, $b_{o}$ represents the model constant, and the model coefficients $b_{i}$, $b_{\mathrm{ii}}$, and $b_{\mathrm{im}}$ are the representatives of the linear, quadratic, and interaction effects of the variables. The data of the designed experiment were analyzed using Design Expert software (Version 8.0.6 Srar‐Ease Inc. Minneapolis, MN, USA) to calculate the expected response. The validity of the statistical experimental design was confirmed through the use of additional confirmation trails.

### 2.6 Purification of the Anthocyanins Extract of *B. kaschgarica* Fruit for In Vivo Activity

**FIGURE 2:**
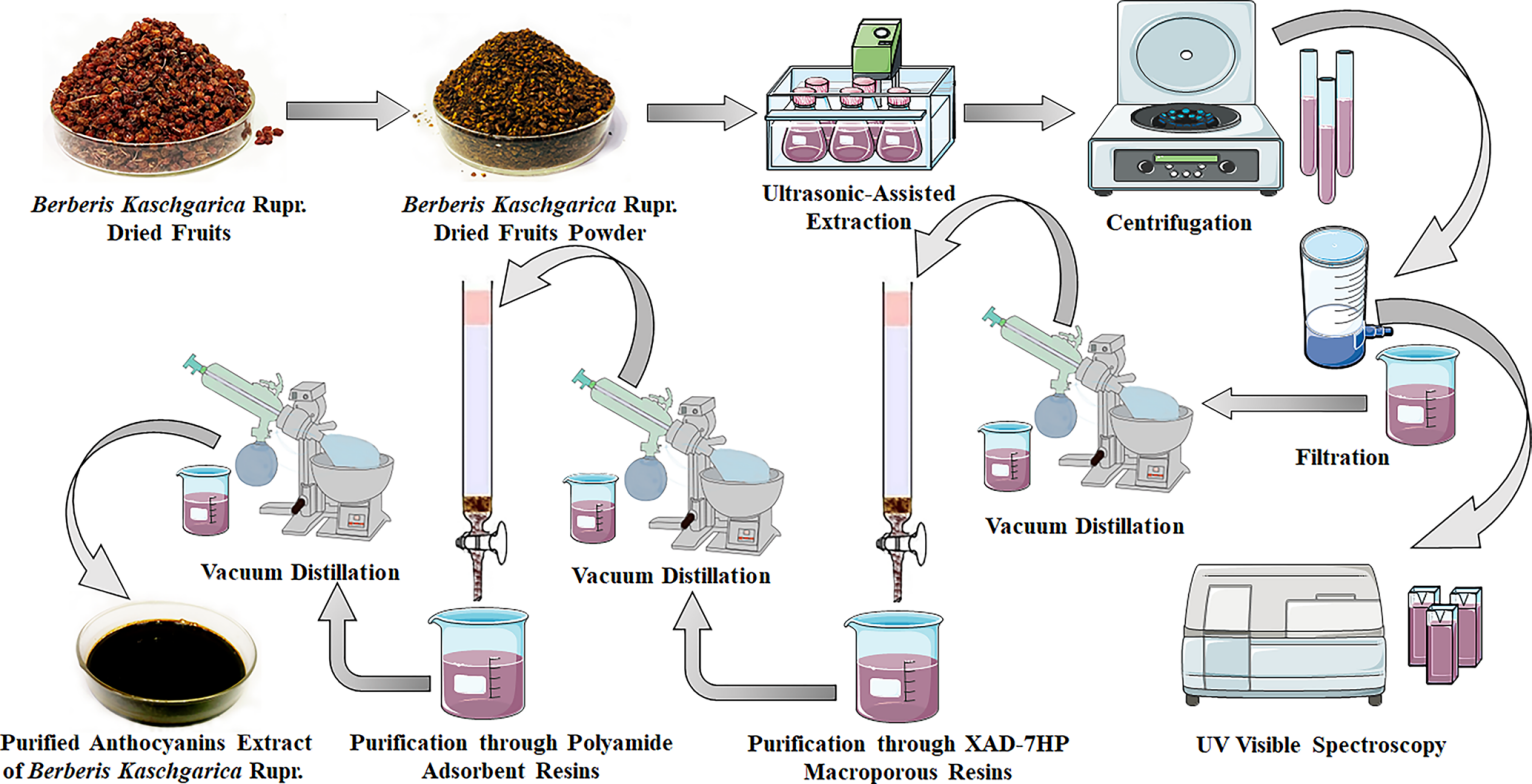
Extraction and purification of anthocyanins from *Berberis kaschgarica* dried fruits.

To determine the in vivo effect of the extract, the anthocyanins from the dried fruit powder were extracted using the determined optimal extraction conditions by UAE. The extract was then filtered through Whatman No.1 filter paper and concentrated under reduced pressure using a rotatory evaporator at 40°C (EYELA N‐1001, EYELA Co. Ltd. Shanghai, China). Two different adsorbent resins were utilized for the purification process. Initially, the macroporous XAD‐7HP resins (Solarbio, Bejiang) and polyamide adsorbent (Solarbio, Bejiang) were soaked separately for 24 h in absolute ethanol at 1:20 (resin/solvent ratio) and transferred to chromatographic glass columns. The columns were washed with distilled water (DW) until the complete removal of ethanol. The XAD‐7HP macroporous resins underwent treatment with 1 M NaOH solution for 24 h, followed by washing with DW until complete removal of the NaOH solution, and then soaked again in 1 M HCL solution for 24 h. Meanwhile, the polyamide column was treated with 5% w/v NaOH solution for 12 h, washed with DW, and treated with 10% acetic acid solution for the next 12 h. After treating both columns with acidic solutions, they were washed with DW until became neutral. Subsequently, the obtained crude extract was loaded into the chromatographic column containing XAD‐7HP macroporous resin, washed with DW twice (1 L each time) to remove the water‐soluble impurities and the anthocyanins were collected with 55% ethanol containing 0.7% HCL by volume. The collected solution of anthocyanins was concentrated until the complete removal of ethanol and loaded into an additional chromatographic column containing polyamide adsorbent. The column was washed with DW twice (1 L each time), and the anthocyanins were eluted with 55% ethanol containing 0.7% HCL by volume. The final purified anthocyanins solution was concentrated under reduced pressure and freeze‐dried. The obtained powder was stored in the dark at −20°C until use. The extraction and purification process is illustrated in Figure 2.

### 2.7 Experimental Animals Protocol

For the experiment 50 SHR rats and 10 Wistar–Kyoto (WKY) rats (11 weeks old), half male (weight 220–220 g), and half female (weight 170–190 g) were purchased from Beijing Weitong Lihua Laboratory, Animal Technology Co. Ltd. (License no: SCXK (Beijing) 2016‐0006) and housed in a specific pathogen‐free (SPF) barrier system within the Animal Research Laboratory of Xinjiang Medical University (license no: SCXY‐(NEW) 2018‐0003). Five rats of the same gender were accommodated in each cage with free access to food and water. The ambient condition of the housing was maintained at a temperature of 22°C ± 2°C and humidity 55% ± 5%. After 1 week (animals aged 12 weeks) the animals were treated with the standard drug and the purified extract of *B. kaschgarica* anthocyanins. The drug and the extract were administered by oral gavage for 12 weeks period. All the animals underwent once weekly body weight measurement.

To assess the in vivo effect of the purified extract of *B. kaschgarica* anthocyanins on hypertensive rats, the rats were randomly divided into six groups with one group comprising WKY rats and the remaining five groups consisting of SHR rats, each group having five male and five female animals. The specific groupings were as follows:

- Negative control group (WKY; *n* = 10): Animals were orally administered 10 mL/kg DW daily for 12 weeks.
- Model group (SHR; *n* = 10): Animals were orally administered 10 mL/kg DW daily for 12 weeks.
- Positive control group (SHR + CAP; *n* = 10): Animals were orally administered captopril at the dose of 12.5 mg/kg daily for 12 weeks.
- *B. kaschgarica* low dose group (SHR + BKRA‐L; *n* = 10): Animals were orally administered with 50 mg/kg of purified extract of *B. kaschgarica* anthocyanins daily for 12 weeks.
- *B. kaschgarica* middle dose group (SHR + BKRA‐M; *n* = 10): Animals were orally administered with 100 mg/kg of purified extract of *B. kaschgarica* anthocyanins daily for 12 weeks.
- *B. kaschgarica* high dose group (SHR + BKRA‐H; *n* = 10): Animals were orally administered with 200 mg/kg of purified extract of *B. kaschgarica* anthocyanins daily for 12 weeks.

#### 2.7.1 Blood Pressure Measurements

Prior to commencing the administration of the therapeutical agents, all the animals underwent 5 days training period of blood pressure measurement, conducted once daily. Following the initiation of the treatment, systolic blood pressure (SBP) and diastolic blood pressure (DBP) were assessed every 2 weeks using a non‐invasive tail cuff BP analyzer (BP‐2000, Visitech System, Apex, NC, USA) until the end of the study. To optimize the detection of the pulse from rat's tail artery, the rats were pre‐warmed for 15 min at 37°C before the experiment. The SBP and DBP values are reported as the average of five consecutive constant measurements.

#### 2.7.2 Measurement of Hemodynamic Indexes

Following 12 weeks of treatment with therapeutic agents, the animals were anesthetized with 2% isoflurane and fixed in a supine position on a dissecting table. A surgical procedure was performed for the exposure of the right common carotid artery. Subsequently, the artery was catheterized using polyethylene catheter (PE‐50) filled with heparinized saline solution and connected to a digital powerlab system (Powerlab/4SP‐ML 750, AD Instruments, USA) via a pressure transducer (TRI 21, Letica Scientific Instruments). After 15 min of stabilizing period heart rate (HR), arterial SBP, and DBP were measured. Next, the catheter was gradually inserted into the left ventricle, followed by 15 min stabilization period. Subsequently, functional variables including left ventricular systolic blood pressure (LVSP), left ventricular end‐diastolic blood pressure (LVEDP), maximum increased rate of ventricular pressure (LV + dp/dtmax), and the rate of decay in ventricular pressure (LV‐dp/dtmax) were measured. The signals were expressed in mmHg and the data were analyzed using LabChart software 7.

#### 2.7.3 Histopathological Examination of Thoracic Aorta and Left Ventricle

Samples of the thoracic aorta and left ventricle were fixed in 4% paraformaldehyde for 48 h and then embedded in paraffin. The embedded samples were then sectioned into slices of 5 μm thickness and stained using hematoxylin and eosin (H&E) staining solution. At least three fields of view from each slice were captured with a light microscope at ×200 magnification (Lecia, Solms, Germany).

#### 2.7.4 Enzyme‐Linked Immunosorbent Assay

After measuring the hemodynamics, blood samples were immediately collected by puncturing the abdominal aorta into blood sample collecting tubes. The collected blood samples were centrifuged at 3000 rpm for 20 min at 4°C and the resulting serum was carefully transferred into separate tubes. Until further analysis, these tubes were stored at −80°C. The serum level concentration of REN, ACE, Ang‐II, angiotensin‐(1–7) (Ang‐(1–7)), NO, and ET‐1 were determined using the specific rat Elisa kits. The REN, ACE, Ang‐II, and Ang‐(1–7) Elisa kits were purchased from Elebscience (Elabscience, Wuhan, China), while the ET‐1 and NO Elisa kits were obtained from Shanghai Elisa Biotech (Shanghai Elisa Biotech Co. Ltd. China) and Beyotime Biotechnology (Beyotime Biotechnology, Shanghai, China), respectively. The assays were performed according to the manufacturer's instructions. The readings of the experiments were measured using the VICTOR‐Nivo Multimode Plate Redder (Perkin Elmer, Beaconsfield, UK).

### 2.8 Data Processing

Data are presented as mean ± SD. Statistical analysis was performed using GraphPad Prism version 8.0 (GraphPad Software, San Diego, CA, USA) and Design Expert 8.0.6 software. One‐way ANOVA followed by the Tukey post hoc test was used to assess significant differences among groups. Significant level denoted as (*p < 0.05, **p < 0.01, ***p < 0.001 vs. SHR group; #*p* < 0.5, ##*p* < 0.01, ###*p* < 0.001 vs. WKY group).

## 3 Results

### 3.1 Single Factor Analysis

#### 3.1.1 Concentration of Solvent

**FIGURE 3:**
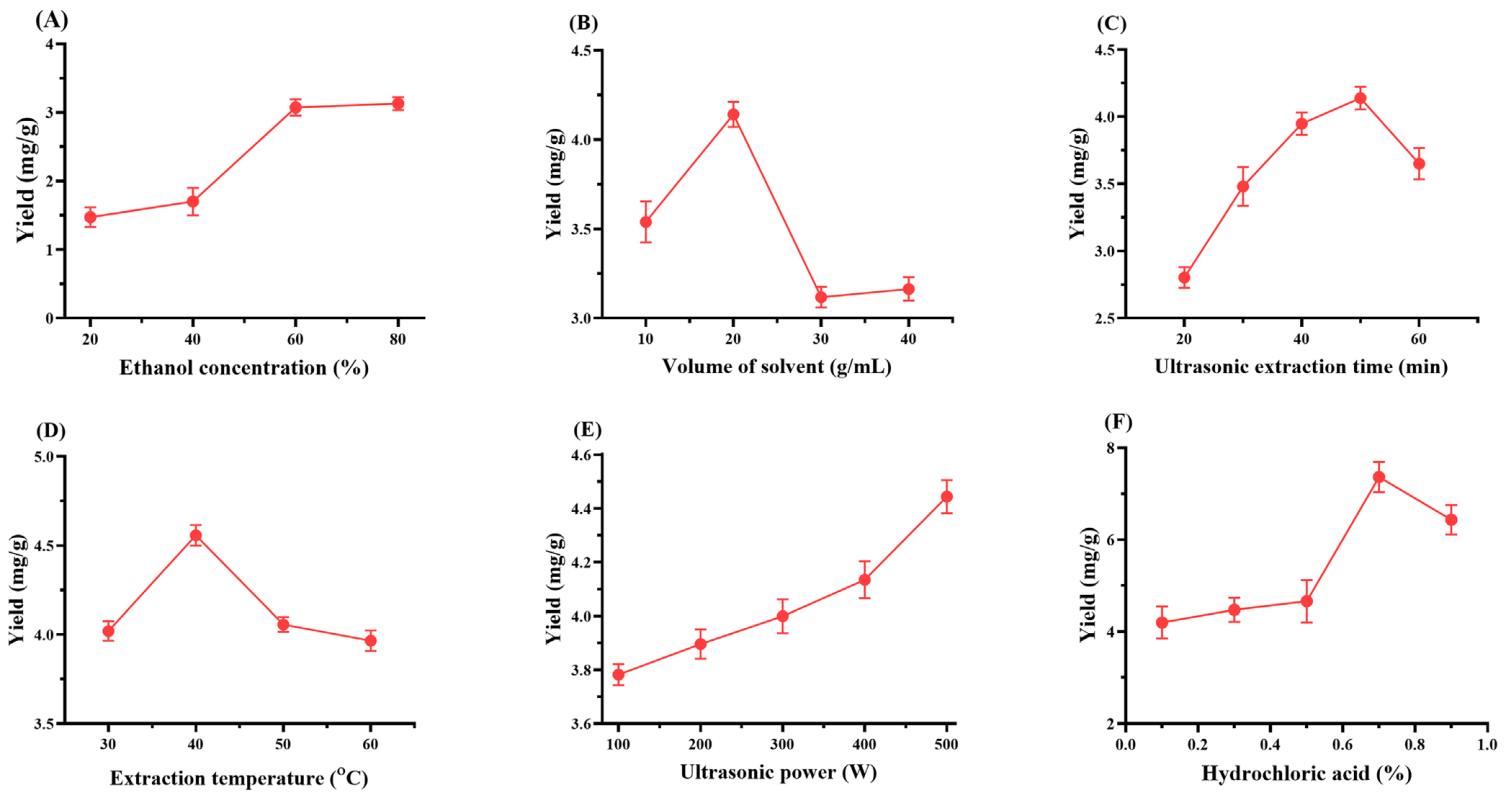
The impact of various extraction parameters on the yield of anthocyanins from *Berberis kaschgarica* dried fruit powder: (A) Effect of ethanol concentration on the yield of anthocyanins. Other factors were fixed as 1% HCL concentration, 1:10 solid‐to‐liquid ratio, 30°C temperature, ultrasonic power 500 W, and 30 min extraction time. (B) Effect of solid‐to‐liquid ratio on the yield of anthocyanins. Other factors were fixed as 1% Hcl concentration, 60% aqueous ethanol solution, 30°C temperature, ultrasonic power 500 W, and 30 min extraction time. (C) Effect of ultrasonic extraction time on the yield of anthocyanins. Other factors were fixed as 1% HCL concentration, 60% aqueous ethanol solution, 1:20 solid‐to‐liquid ratio, 30°C temperature, and ultrasonic power 500 W. (D) Effect of temperature on the yield of anthocyanins. Other factors were fixed as 1% HCL concentration, 60% aqueous ethanol solution, 1:20 solid‐to‐liquid ratio, 50 min extraction time, and ultrasonic power 500 W. (E) Effect of the ultrasonic power on the yield of anthocyanins. Other factors were fixed as 1% HCL concentration, 60% aqueous ethanol solution, 1:20 solid‐to‐liquid ratio, 50 min extraction time, and 40°C temperature. (F) Effect of HCL concentration on the yield of anthocyanins. Other factors were fixed as 60% aqueous ethanol solution, 1:20 solid‐to‐liquid ratio, 50 min extraction time, 40°C temperature, and ultrasonic power 500 W.

The concentration of solvent is a major and crucial step for extraction of the bioactive compounds from plant materials (Routray and Orsat 2012). In this study, 1% HCL‐acidified aqueous ethanol solution with varying ethanol concentrations (20%, 40%, 60%, and 80%) was utilized for extracting anthocyanins from *B. kaschgarica* dried fruit powder. The solid‐to‐liquid ratio was maintained at 1:10 with a constant temperature of 30°C and ultrasonic power set at 500 W for 30 min duration. The experimental findings revealed a direct relationship between ethanol concentration and anthocyanin content, as illustrated in Figure 3A. Notably, the use of 60% and 80% ethanol concentrations resulted in extracted anthocyanin yields of 3.074 and 3.13 mg/g, respectively, with minimal difference observed between them. Considering both cost effectiveness and yield the 60% acidified ethanolic solution was selected for further experiments.

#### 3.1.2 Volume of Solvent

The volume of solvent plays a pivotal role in the extraction process, as it directly influences the solubility of constituents (Li, Chen, and Yao 2005). Typically, a large volume of solvent facilitates easy dissolution of compounds; however, excessive use of solvent may lead to wastage. Conversely, employing a smaller volume of solvent may result in a lower extraction yield (Valachovic, Pechova, and Mason 2001). Hence, it is important to identify an appropriate solid‐to‐liquid ratio to optimize extraction efficiency.

To achieve the best extraction condition for anthocyanins from *B. kaschgarica* dried fruit powder four different solid‐to‐liquid ratios (1:10, 1:20, 1:30, and 1:40) were investigated. 60% aqueous‐ethanolic solution (containing 1% HCL) was used with a constant temperature was 30°C and ultrasonic power set at 500 W for 30 min. The experimental results revealed a substantial increase in anthocyanin yield at a solid‐to‐liquid ratio of 1:20 (4.14 mg/mL) as depicted in Figure 3B. Therefore, for further experiments, the solid‐to‐liquid ratio of 1:20 was adopted.

#### 3.1.3 Extraction Time

The duration of the extraction significantly influences the yield of extracted compounds (Kumar, Srivastav, and Sharanagat 2021). To determine the optimal ultrasonic extraction time for anthocyanins from *B. kaschgarica* dried fruit powder, extraction was conducted at various time intervals ranging from 20 to 60 min. A 60% aqueous‐ethanolic solution with 1% HCL concentration was used as extraction solvent while maintaining solid‐to‐liquid ratio of 1:20, temperature 30°C, and ultrasonic power at 500 W. As illustrated in Figure 3C, an increase in the yield of anthocyanins was observed with an increase in ultrasonic extraction time. Notably at 50‐min time point, the extraction yield peaked at 4.13 mg/mL of anthocyanins. Subsequently, at 60‐min time point decrease in anthocyanin yield was noted. Therefore, the ultrasonic extraction time of 50 min was determined as the optimal duration for the experiment.

#### 3.1.4 Extraction Temperature

Similar to other factors temperature significantly influences extraction yield, particularly for heat‐sensitive compounds as it affects solvent diffusion rates and mass transfer intensification, which in turn influences the dissolution of targeted compounds (Dong et al. 2010). However, it is essential to consider that high temperatures may lead to the decomposition of thermally labile components, such as anthocyanins.

In this study, various temperatures ranging from 30°C to 60°C were explored to determine the ideal extraction temperature for anthocyanins. Solid‐to‐liquid ratio of 1:20 was maintained and 60% aqueous ethanolic solution containing 1% HCL was used as extraction solvent. Additionally, the ultrasonic power was set at 500 W, and the extraction time was 50 min.

As depicted in Figure 3D, the highest yield of extracted anthocyanins was observed at 40°C, reaching 4.55 mg/mL. However, further increases in temperature resulted in a decline in anthocyanin production. Based on the obtained results 40°C was selected as standard extraction temperature for subsequent experiments.

#### 3.1.5 Ultrasonic Power

The ultrasonic power utilized during extraction plays a crucial role in determining extraction efficiency. The results obtained for other parameters such as solid‐to‐liquid ratio 1:20, temperature 40°C, extraction time 50 min, and ethanol concentration 60% containing 1% HCL were used, and the extraction was optimized for the ultrasonic power at 5 different points (100–500 W). As illustrated in Figure 3E, an increase in ultrasonic power corresponded to an increase in anthocyanin yield. Notably, the highest yield was achieved at 500 W, making it the optimal ultrasonic power for the extraction.

#### 3.1.6 HCL Volume

All other parameters were set to their optimized level, that is, solid‐to‐liquid ratio 1:20, temperature 40°C, extraction time 50 min, ultrasonic power 500 W, and ethanol concentration 60%. Then different volumes of HCL ranging from 0.1% to 0.9% were tested during the extraction process. It was found that the solvent containing 0.7% HCL exhibited the highest concentration of anthocyanins, yielding 7.36 mg/mL (Figure 3F). Therefore 0.7% HCL volume was selected as the optimal volume concentration for HCL.

### 3.2 Optimization of the Anthocyanins Yield Through Response Surface Methodology

The yield of anthocyanins of *B. kaschgarica* dried fruit powder was further optimized through the RSM approach. The impact of four key factors, that is, ethanol concentration (*X*_1_), solid‐to‐liquid ratio (*X*_2_), extraction time (*X*_3_), and HCL volume (*X*_4_) were assessed in the designed experiments to maximize the anthocyanins yield from *B. kaschgarica* dried fruit powder by UAE. The experimental conditions were set with fixed ultrasonic power (500 W) and temperature (40°) to ensure the optimal extraction without compromising the integrity of anthocyanins. High temperatures can easily hydrolyze or oxidize anthocyanins which may affect the yield (Dzah et al. 2020), while with high ultrasonic power larger size resonant bubbles are produced which results in increasing diffusivity and improved extraction yield (Maran and Priya 2014).

The experiment design consisted of 4 factors at 3 levels each, detailed in Table 2 along with their coded levels and corresponding anthocyanins yield. The extracted anthocyanins yield ranged from 4.7 to 7.3 mg/g. The condition yielding maximum anthocyanins yield was demonstrated to be: *X*_1_ = 60%, *X*_2_ = 1:20, *X*_3_ = 50 min, and *X*_4_ = 0.7%. The relationship between the test variables and the response variables (yield) was established using multiple regression analysis, resulting in the following secondary‐order polynomial equation:

$$\begin{array}{cc} Y=\; & -56.61834+0.071432X_{1}+0.81887X_{2}\\ & +0.43497X_{3}+115.5413X_{4}-4.50\times 1.0^{-5}X_{1}X_{2}\\ & +4.46\times 10^{-3}X_{1}X_{3}+0.030250X_{1}X_{4}-7.74\times 10^{-3}X_{2}X_{3}\\ & +0.27850X_{2}X_{4}-0.598X_{3}X_{4}-4.69\times 1.0^{-3}X_{1}^{2}\\ & -0.014878X_{2}^{2}-5.88\times 1.0^{-4}X_{3}^{2}-74.4325X_{4}^{2} \end{array}$$

**TABLE 2:** Response surface design and the experimental data.

Test set	Coded levels				Anthocyanin yield mg/g
	(*X*_1_)	(*X*_2_)	(*X*_3_)	(*X*_4_)	
1	0	1	−1	0	6.784
2	0	−1	1	0	7.036
3	0	0	1	1	6.988
4	0	0	0	0	7.088
5	0	0	0	0	6.983
6	0	0	0	0	7.254
7	0	0	0	0	7.389
8	0	0	1	−1	6.628
9	−1	1	0	0	7.306
10	−1	−1	0	0	7.032
11	1	1	0	0	5.718
12	−1	0	−1	0	7.132
13	1	0	−1	0	5.610
14	0	1	0	1	6.797
15	1	0	0	−1	4.704
16	−1	0	0	1	6.471
17	−1	0	1	0	7.312
18	0	1	0	−1	5.371
19	0	0	−1	−1	5.323
20	1	−1	0	0	5.444
21	0	0	0	0	7.093
22	0	1	1	0	7.175
23	0	−1	−1	0	5.871
24	1	0	1	0	6.682
25	−1	0	0	−1	6.088
26	0	−1	0	−1	5.584
27	0	−1	0	1	6.453
28	0	0	−1	1	6.879
29	1	0	0	1	6.297

The analysis of variance (ANOVA) for the regression equation reveals that both linear and quadratic terms were highly significant (*p* < 0.001) indicating the robustness of the model (Table 3). Additionally, the lack of fit was found to be not significant (*P* > 0.05), suggesting that the model adequately fits the experimental data. The adjusted *R* ^*2*^ value (0.9197) for the equation is particularly close to 1, indicating a strong correlation between the predicted and observed values. This suggests that the model is credible and can explain 91.97% of the variability of the experimental data. The order of influence of experimental factors on the anthocyanin contents of *B. kaschgarica* dried fruit powder was as follows: ethanol concentration (*X*_1_) > HCL volume (*X*_4_) > extraction time (*X*_3_) > solid‐to‐liquid ratio (*X*_2_).

**TABLE 3:** Analysis of variance (ANOVA) for the regression equation.

SD	SS	DF	MS	*F* value	*p*	Significance
Model	14.85	14	1.06	23.91	< 0.0001	**
$X_{1}$	3.94	1	3.94	88.85	< 0.0001	**
$X_{2}$	0.25	1	0.25	5.69	0.0318	*
$X_{3}$	1.49	1	1.49	33.49	< 0.0001	**
$X_{4}$	3.19	1	3.19	71.92	< 0.0001	**
$X_{1}X_{2}$	2.025 × 10^−5^	1	2.025 × 10^−5^	4.565 × 10^−4^	0.9833	
$X_{1}X_{3}$	0.20	1	0.20	4.48	0.0526	
$X_{1}X_{4}$	0.37	1	0.37	8.25	0.0123	*
$X_{2}X_{3}$	0.15	1	0.15	3.38	0.0874	
$X_{2}X_{4}$	0.078	1	0.078	1.75	0.2072	
$X_{3}X_{4}$	0.36	1	0.36	8.06	0.0131	*
$X_{1}^{2}$	1.43	1	1.43	32.14	< 0.0001	**
$X_{2}^{2}$	0.90	1	0.90	20.23	0.0005	**
$X_{3}^{2}$	1.402 × 10^−3^	1	1.402 × 10^−3^	0.032	0.8615	
$X_{4}^{2}$	3.59	1	3.59	81.02	< 0.0001	**
Residual	0.62	14	0.044			
Lack of fit	0.52	10	0.052	2.03	0.2585	
Pure error	0.10	4	0.026			
Sum	15.47	28				
			*R* ^2^ = 0.9599	*R* ^2^_Adj_ = 0.9197		

* *p* < 0.05.

** *p* < 0.01.

**FIGURE 4:**
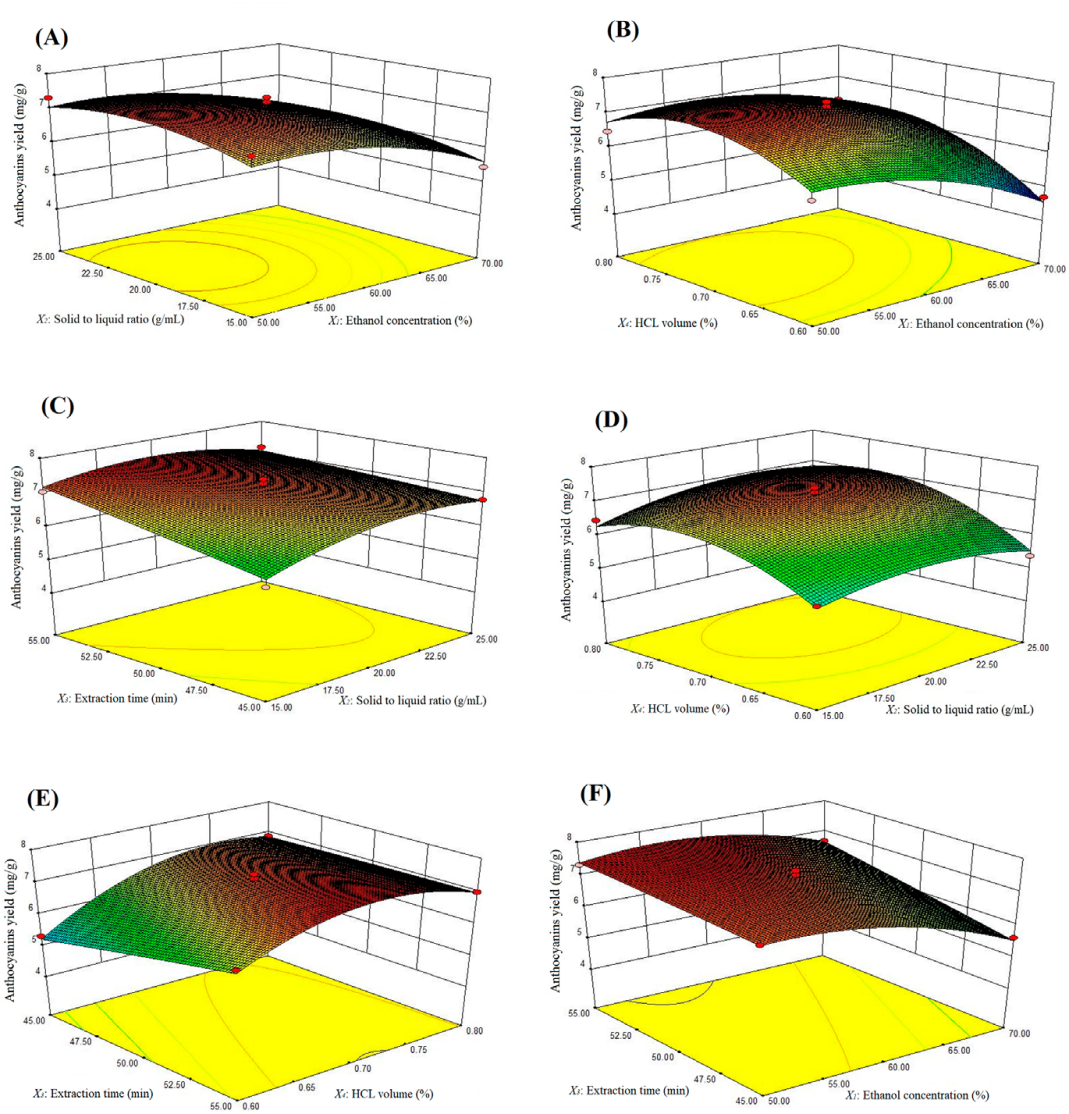
Response surface graphs representing the effects of the four experimental factors on the yield of anthocyanins from *B. kaschgarica* dried fruit powder: (A) Ethanol concentration (*X*_1_) and solid‐to‐liquid ratio (*X*_2_). (B) Ethanol concentration (*X*_1_) and HCL volume (*X*_4_). (C) Solid‐to‐liquid ratio (*X*_2_) and extraction time (*X*_3_). (D) Solid‐to‐liquid ratio (*X*_2_) and HCL volume (*X*_4_). (E) Extraction time (*X*_3_) and HCL volume (*X*_4_). (F) Ethanol concentration (*X*_1_) and extraction time (*X*_3_).

To visualize the impact of factors and their interactions on the anthocyanin extraction from *B. kaschgarica* dried fruit powder and determine the optimal level range of each factor, three‐dimensional response surface plots were generated using Design Expert 8.0.6 software (Figure 4). Increased ethanol concentration (*X*_1_) and HCL volume (*X*_2_) initially increased anthocyanins yield up to a certain level (Figure 4B). Similarly, elevating HCL volume (*X*_4_) and the extraction time (*X*_3_) initially boosted anthocyanins yield followed by a gradual decline (Figure 4E). Furthermore, higher ethanol concentration (*X*_1_) and extraction time (*X*_3_) notably enhanced anthocyanin yield (Figure 4F). However, the interaction between other factors showed no significant effect on the anthocyanins extraction yield from *B. kaschgarica* dried fruit powder.

By solving the regression equation, the optimal values were obtained for the selected variables. Using Design Expert software, the optimal conditions for anthocyanin extraction from *B. kaschgarica* dried fruit powder were determined to be 56.5% ethanol volume, 1:19.74 solid‐to‐liquid ratio, 55 min extraction time, and 0.71% HCL volume. Under these conditions, the extracted content of anthocyanins reached 7.569 mg/g. Considering the practical operation feasibility, these factors were modified to 55% ethanol volume, 1:20 solid–liquid ratio, 55 min extraction time, and 0.7% HCL volume. Under these adjusted conditions, tests were performed in triplicate, yielding a mean value of 7.524 mg/g which was closer to the predicted value. The experimental results clearly show the fitness of the regression model to the experimental data which means that the model is accurate and adequate for the extraction of anthocyanins from *B. kaschgarica* dried fruit powder.

Furthermore, after the purification process, the anthocyanin content reached 89.76 mg/g significantly higher than the unpurified extract. This result indicates that the macroporous XAD‐7HP resins and polyamide adsorbents effectively retained anthocyanins, enhancing phytochemical purity and offering a cost‐effective purification method.

### 3.3 Effect of the Purified Extract of *B. kaschgarica* Anthocyanins on the Blood Pressure of SHR Rats

**FIGURE 5:**
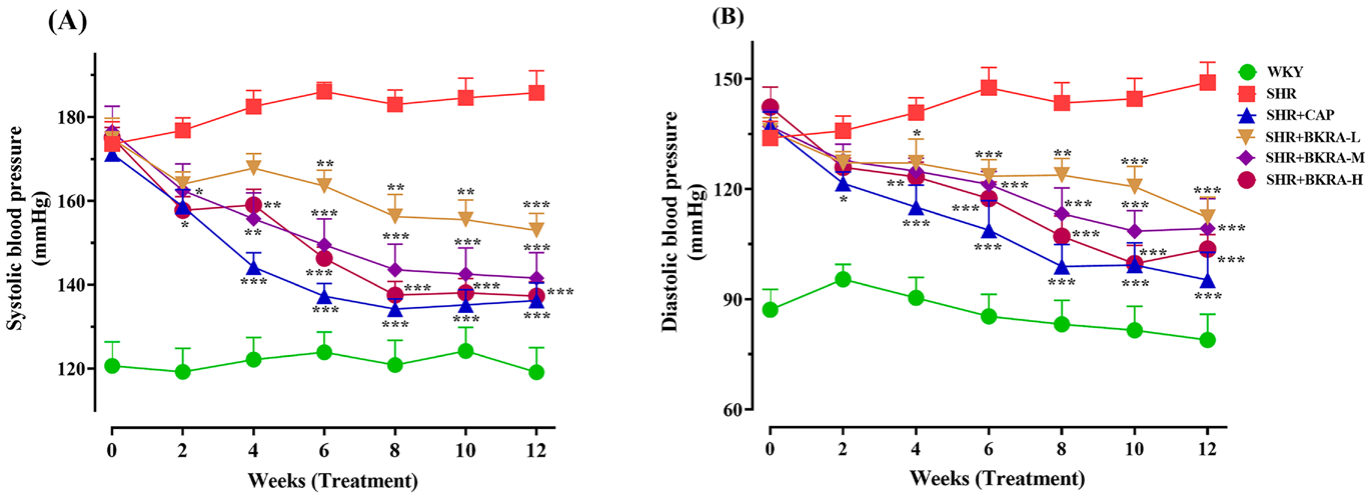
(A) Systolic blood pressure (SBP); (B) Diastolic blood pressure (DBP) during 12 weeks of treatment of the Wistar–Kyoto (WKY) rats and spontaneously hypertensive (SHR) rats groups. WKY (distilled water), SHR group (distilled water), SHR + CAP (12.5 mg/kg of captopril), SHR + BKRA‐L (50 mg/kg of purified extract of *Berberis kaschgarica* anthocyanins), SHR + BKRA‐M (100 mg/kg of purified extract of *B. kaschgarica* anthocyanins), and SHR + BKRA‐H (200 mg/kg of purified extract of *B. kaschgarica* anthocyanins) were administrated by oral gavage for 12 weeks. Each group contains 10 rats. The data are presented as mean ± standard deviation. One‐way analysis of variance (ANOVA) followed by Tukey's post hoc test, significance difference: *p < 0.05, **p < 0.01, ***p < 0.001 versus SHR group.

The SBP and DBP of each group were measured at the beginning of the study (week 0) and constantly every 2 weeks until the end of the experiment. At the beginning of the experiment, there was not any significant difference in the SBP among all the SHR rat groups. However, as the treatment commenced, by week 4 the mean SBP of SHR + CAP, SHR + BKRA‐M, and SHR + BKRA‐H groups had decreased to approximately 79%, 85%, and 86%, respectively, compared to the SHR model group. Subsequently, by week 6 a significant reduction in the SBP was observed across all treated groups in a dose‐dependent manner, compared with the SHR model group as presented in Figure 5A.

Regarding DBP, similar to SBP, at the beginning, no statistically significant was observed among all the SHR rat groups. However, by week 4, a downward trend in DBP was evident, with a significant reduction noted in the SHR + CAP and BKRA treated groups compared to the SHR group. By week 12, the DBP of the SHR + CAP, SHR + BKRA‐L, SHR + BKRA‐M, and SHR + BKRA‐H groups had significantly decreased by 36%, 24%, 27%, and 30%, respectively in compression with the SHR model group (Figure 5B).

### 3.4 Hemodynamic Changes

**FIGURE 6:**
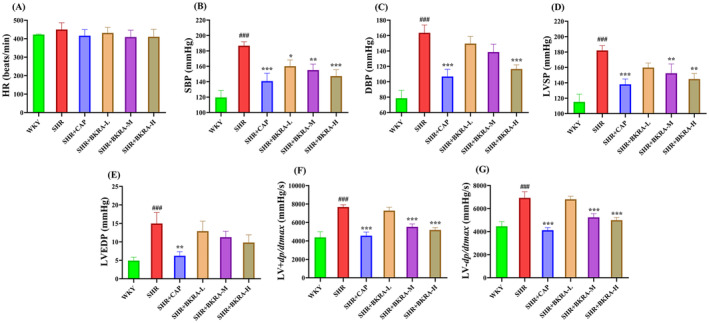
Hemodynamic parameters: (A) heart rate (HR); (B) systolic blood pressure (SBP); (C) diastolic blood pressure (DBP); (D) left ventricular systolic blood pressure (LVSP); (E) left ventricular end‐diastolic blood pressure (LVEDP); (F) maximum increased rate of ventricular pressure (LV + dp/dtmax); and (G) rate of decay in ventricular pressure (LV‐dp/dtmax) after 12 weeks of treatment of Wistar–Kyoto (WKY) rats and spontaneously hypertensive (SHR) rats groups. WKY (distilled water), SHR group (distilled water), SHR + CAP (12.5 mg/kg of captopril), SHR + BKRA‐L (50 mg/kg of purified extract of *B. kaschgarica* anthocyanins), SHR + BKRA‐M (100 mg/kg of purified extract of *B. kaschgarica* anthocyanins), and SHR + BKRA‐H (200 mg/kg of purified extract of *B. kaschgarica* anthocyanins) were administrated by oral gavage for 12 weeks. Each group contains 10 rats. The data are presented as mean ± standard deviation. One‐way analysis of variance (ANOVA) followed by Tukey's post hoc test, significance difference: ### *p* < 0.001 versus WKY group and *p < 0.05, **p < 0.01, ***p < 0.001 versus SHR group.

We investigate the protective effect of purified extract of *B. kaschgarica* anthocyanins on left ventricular cardiac functions. The hemodynamic parameters were evaluated through ventricular catheterization after 12 weeks of anthocyanin extract administration. In comparison with the WKY group, both SBP and DBP were significantly elevated in the SHR group (Figure 6A,B). However, significant reductions in SBP (< 155 mmHg) were observed across all the groups compared to the SHR group (Figure 6A), while only the SHR + CAP (106 mmHg) and SHR + BKRA‐H (116 mmHg) treated groups showed statistically significant reduction compared to the SHR group (Figure 6B).

To evaluate the degree of left ventricular response post‐treatment with the purified extract of *B. kaschgarica* anthocyanins, left ventricular pressure was measured. While the SHR group exhibited significantly increased LVSP, LVEDP, LV + dp/dtmax, and LV‐dp/dtmax compared to the WKY group (*p* < 0.001), treatment groups showed a significant reduction in LVSP (< 150 mmHg) except SHR + KBRA‐L group (160 mmHg) compared to the SHR group (182 mmHg; Figure 6C). Moreover, a nearly two‐fold increase in the LVEDP, indicative of left ventricular dysfunction, was observed in the SHR group in comparison to the WKY group, while a significant reduction was observed in the treatment groups in dose‐dependent manner (Figure 6D).

LV + dp/dtmax and LV‐dp/dtmax are profound indicators reflecting changes in cardiac contractility (Shaikh, Bhatt, and Barve 2019). An extensive increase in both parameters was noted in the SHR group versus the WKY group, potentially leading to myocardial contractility dysfunction. However, treated with purified extract of *B. kaschgarica* anthocyanins significantly reduced the values of these parameters (Figure 6E,F) compared to the SHR group, suggesting the preventive effect of the treatment on the left ventricular contractility dysfunction.

### 3.5 Effect of the Purified Extract of *B. kaschgarica* Anthocyanins on the Left Ventricular and Aorta Histology

**FIGURE 7:**
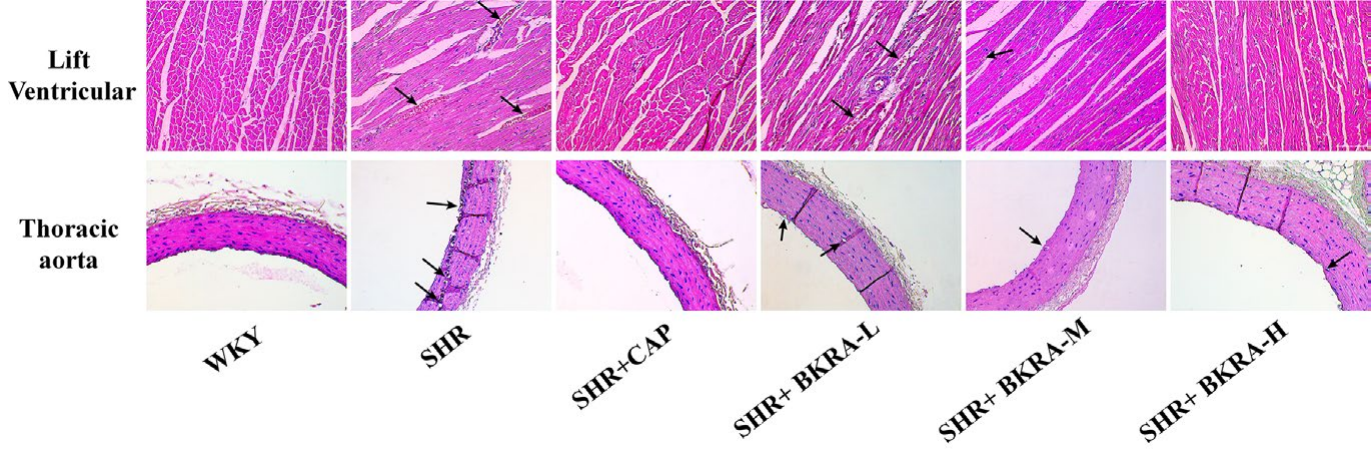
H&E staining of the left ventricular and thoracic aorta (200×) after 12 weeks of treatment of Wistar–Kyoto (WKY) rats and spontaneously hypertensive (SHR) rats groups. WKY group (distilled water), SHR group (distilled water), SHR + CAP group (12.5 mg/kg of captopril), SHR + BKRA‐L (50 mg/kg body of purified extract of *B. kaschgarica* anthocyanins), SHR + BKRA‐M (100 mg/kg of purified extract of *B. kaschgarica* anthocyanins), and SHR + BKRA‐H (200 mg/kg of purified extract of *B. kaschgarica* anthocyanins) were administered for 12 weeks by oral gavage. Each group contains 10 rats.

The histological examination of the left ventricular and aorta tissues was conducted using H&E staining across the WKY, SHR, SHR + CAP, SHR + BKRA‐L, SHR + BKRA‐M, and SHR + BKRA‐H groups (Figure 7). In the SHR group degeneration and hypertrophy were observed in the myocardia and vascular smooth muscles compared to the WKY group. However, in the SHR + CAP, SHR + BKRA‐M, and SHR + BKRA‐H groups alleviated degeneration and hypertrophy were observed in the targeted tissues compared to the SHR group.

### 3.6 Effect of the Purified Extract of *B. kaschgarica* Anthocyanins on Serum REN, ACE, Ang‐II, Ang‐(1–7), ET‐1, and NO Concentration

REN is the initial key enzyme of RAS, secreted by juxtaglomerular cells of kidneys to the circulatory system which cleaves the angiotensinogen into Ang‐I that is further converted to Ang‐II in the lungs by ACE (Atlas 2007; Hsueh 1984). ACE plays a key role in vasoconstriction by increasing Ang‐II production and at the same time, it also degrades several vasodilating peptides including Ang‐(1–7) which leads to hypertension (Nehme et al. 2019). Similarly, the ET‐1 and NO produced by the vascular endothelial cells play an important role in the regulation of vascular contractility (Kostov 2021).

**FIGURE 8:**
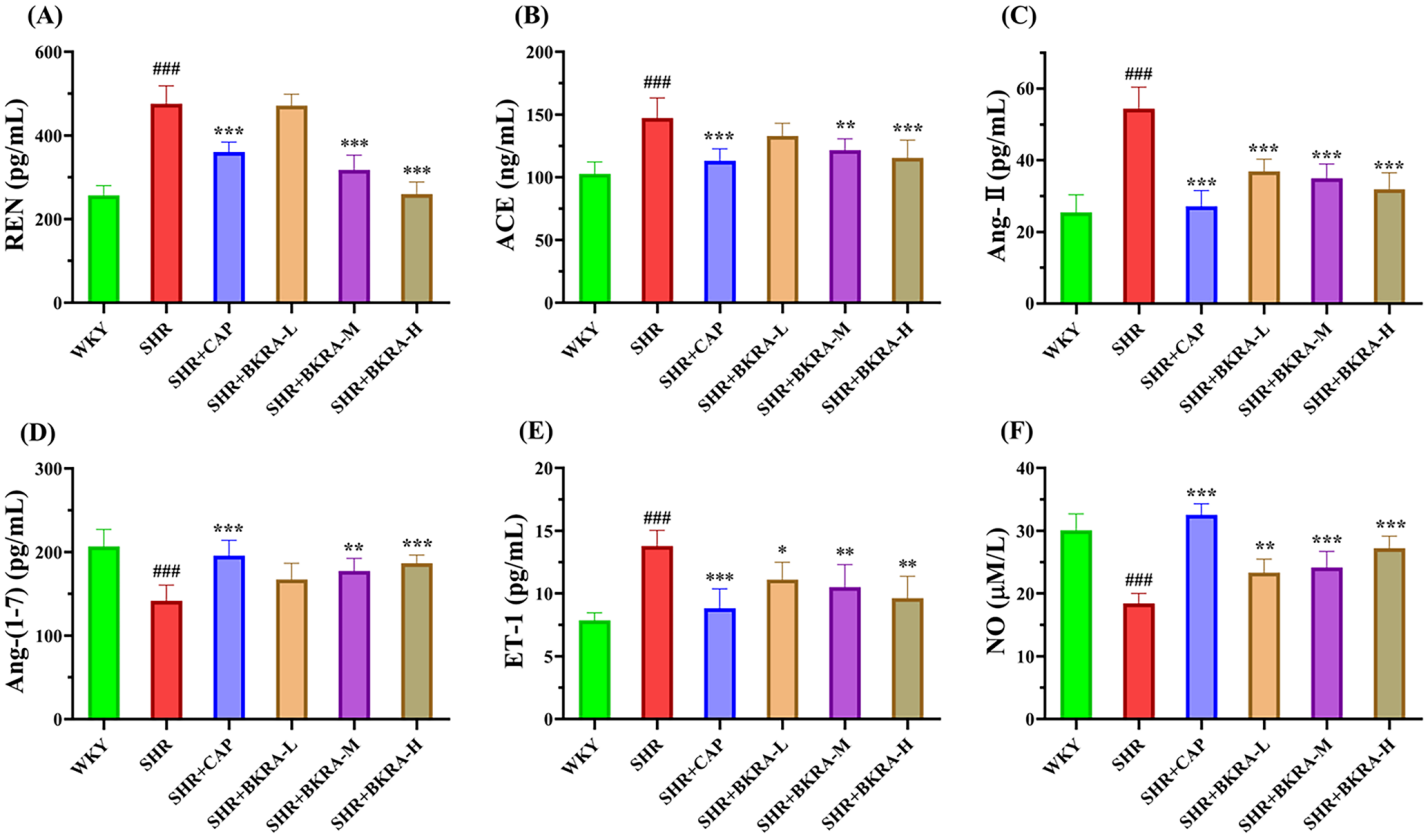
Serum level of (A) renin (REN); (B) angiotensin‐converting enzyme (ACE); (C) angiotensin II (Ang‐II); (D) angiotensin‐(1–7) (Ang‐(1–7)); (E) Endothelin‐1 (ET‐1); and (F) nitric oxide (NO) after 12 weeks of treatment of Wistar–Kyoto (WKY) rats and spontaneously hypertensive (SHR) rats groups. WKY group (distilled water), SHR group (distilled water), SHR + CAP group (12.5 mg/kg of captopril), SHR + BKRA‐L (50 mg/kg body of purified extract of *B. kaschgarica* anthocyanins), SHR + BKRA‐M (100 mg/kg of purified extract of *B. kaschgarica* anthocyanins), and SHR + BKRA‐H (200 mg/kg of purified extract of *B. kaschgarica* anthocyanins) were administered for 12 weeks by oral gavage. Each group contains 10 rats. The data are presented as mean ± standard deviation. One‐way analysis of variance (ANOVA) followed by Tukey's post hoc test, significance difference: ### *p* < 0.001 versus WKY group and *p < 0.05, **p < 0.01, ***p < 0.001 versus SHR group.

In the current experiment, as shown in Figure 8A the serum concentration of REN in the SHR group significantly increased compared to the WKY group (*p* < 0.001), while the REN concentration in the SHR + CAP (360 pg/mL), SHR + BKRA‐M (317 pg/mL), and SHR + BKRA‐H (260 pg/mL) were noticed significantly reduced in comparison to SHR group (Figure 8A). Similarly, the concentration of ACE in serum of the SHR group increased significantly (147 ng/mL) in comparison with the WKY group, although in the SHR + CAP (113 ng/mL), SHR + BKRA‐M (121 ng/mL), and SHR + BKRA‐H (115 ng/mL) groups, the concentration of ACE was found significantly lower compared to SHR group (Figure 8B). On the other hand, the serum level of Ang‐II in the SHR group was found almost 53% higher than the WKY group (Figure 8C). The SHR + CAP group and the groups treated with the extract were found with a significantly decreased level of serum Ang‐II concentration than the SHR group (50% CAP, 32% BKRA‐L, 35% BKRA‐M, and 41% BKRA‐H; Figure 8C). Furthermore, it was also noticed that the serum concentration of Ang‐(1–7) was significantly lower (38%) in the SHR group in comparison with the WKY group (Figure 8D), while the level of Ang‐(1–7) in SHR + CAP, SHR + BKRA‐M, and SHR + BKRA‐H groups were found significantly higher as compared to the SHR group (38%, 25%, and 31%, respectively; Figure 8D). Alternatively, in the SHR group, the serum concentration of ED‐1 was found to double (13 pg/mL) than in the WKY group, although in the SHR + CAP (8 pg/mL) and treated groups of purified extract of *B. kaschgarica* anthocyanins (*p* < 0.01) was significantly reduced as compared to the SHR group (Figure 8E). In addition, it was also noticed that the serum level of NO was significantly reduced in the SHR group (18 μM/L) as compared to the WKY group, while in the SHR‐treated groups (CAP, BKRA‐L, BKRA‐M, and BKRA‐H) it was significantly increased (*p* < 0.001) as compared to SHR group (Figure 8F). These results indicate a clear role of the purified extract of *B. kaschgarica* anthocyanins in reducing hypertension by interfering with the activity of the RAS system and vascular endothelial factors.

## 4 Discussion

The central theme of the current study was to establish the best conditions for UAE extraction of anthocyanins from *B. kaschgarica* dried fruit powder and to evaluate its potential antihypertensive effects in SHR rats. UAE is one of the most effective techniques for extracting phytochemicals from natural products (López et al. 2018). In ultrasonic extraction, the cell wall rupture occurs due to cavitation effects that result in the increasing of the contact area between the solvent and solid phase (Pingret et al. 2012). It has also been reported as a faster and less energy‐consuming method of extraction in comparison with other conventional methods (Jacotet‐Navarro et al. 2016). Furthermore, less consumption of solvent and yielding a high purity level of extract made the process more reliable (Wang and Weller 2006). To establish the best UAE parameters, we first determined the optimal extraction point for each parameter through single‐factor experiments and then used the optimum extraction points together for the UAE process to maximize anthocyanin yield. During the extraction of phenolic compounds, ethanol always remains the first choice for extraction due to its high affinity (Ramić et al. 2015), affordability, and is categorized as GRAS (generally recognized as safe) solvent (Rodrigues et al. 2015). Increasing ethanol concentration to a certain level will increase the yield of the extracted compounds but after that, the higher concentrations have been noticed with a negative effect on the yield of the extracting compounds. This effect may be caused by increasing the solubility and diffusivity of the phenolic compounds with a decrease in the dielectric constant of the solvent due to higher ethanol concentration (Kumar, Srivastav, and Sharanagat 2021). Therefore, in the current study, the selected concentration of ethanol was 60% which was found effective in extracting a high yield of anthocyanins as well as cost‐effective for the experiment. For good extraction practices not only the concentration of the solvent is important, but it is also mandatory to select a well‐match solvent for the extracting compounds (Lupacchini et al. 2017). For instance, Boulekbache‐Makhlouf et al. (2013) reported that acidified organic solvent was found more effective for the extraction of anthocyanins from eggplant peels. Similarly, Jiang, Yang, and Shi (2017), clarified in their work that anthocyanins are more stable in an acidified medium, and acidified ethanolic solvent is the best option for extracting anthocyanins. Therefore, in our experiment, different HCL concentrations were examined and 0.7% acidified ethanolic solution of HCL was found with the highest extraction yield of anthocyanins. Regarding solvent volume, in the current experiment, a 1:20 g/mL solid‐to‐liquid ratio was found with the highest yield of anthocyanins (Figure 3B). The volume of solvent plays an important role in the extraction process (Kumar, Srivastav, and Sharanagat 2021). Many studies have shown that the yield of anthocyanins can be affected by the solid‐to‐liquid ratio (Xue et al. 2021b). The reason behind this phenomenon is that with the increase in solvent concentration the concentration gradient increases, and the solid‐to‐liquid contact area also increases which may result in improving the diffusion of the anthocyanins from the solid phase and yield a high amount of anthocyanins (Mazza et al. 2019). In this study, we noticed a downward trend in the anthocyanin yield after increasing the solid‐to‐liquid ratio from 1:20 g/mL. The optimal solid‐to‐liquid ratio for maximum extraction of anthocyanins varies on various factors such as source materials and processing parameters before extraction. Recently in a study, it was noticed that the yield of anthocyanins was increased up to a certain level while increasing the solid‐to‐liquid ratio, due to increased concentration gradient, but suddenly decreased as the ratio was further increased (Blackhall et al. 2018). He et al. (2016) also reported similar results for the solvent volume in UAE of anthocyanins from blueberry wine pomace. The extraction time in UAE may also affect the yield of anthocyanins. The extraction time is directly associated with the power value supplied by the ultrasound and the mass of the sample (Strieder, Silva, and Meireles 2019). If the sample is exposed for a much longer time it may result in the degradation of the active ingredients while in less time exposure it may not be extracted completely (Roriz et al. 2017). In the current experiment, until 50 min of extraction time, an increase in the yield of anthocyanins was observed but after that, the level suddenly starts to fall, which means that increasing time results in degradation of anthocyanins. Similar results were also reported by others (Espada‐Bellido et al. 2017). During UAE the temperature also affects the yield of the anthocyanins. Anthocyanins can easily hydrolyze or oxidize with rise in temperature (Dzah et al. 2020). In the current experiment, a downtrend was noticed as the temperature increased from 40°C. Similar results were presented by Xue et al. (2021a), during the extraction of anthocyanins from cranberry. Another factor in ultrasonic extraction is ultrasonic power which plays an important role in the extraction of the bioactive compounds from the source. The increase in the ultrasonic power to a certain level boosts the yield of the extract (Xu et al. 2014; Wang et al. 2015; Chemat et al. 2017a). This phenomenon can be explained because of the increasing power that directly increases the violent cavitation bubbles collapse (Wen, Zeng, and Mai 2022). The ultrasonic wave is proportional to the size of the resonant bubble, as the bubble size increases, its effect on implosion also intensifies which causes pore formation and fragmentation in the tissues resulting in increasing the diffusivity and improved extraction yield (Maran and Priya 2014). Similar effects have been noticed in the current experiment, as the ultrasonic power increased the yield of anthocyanins increased.

RSM is the technique to understand the interaction and influence of different experimental factors such as time, temperature, reagents concentration, solid–liquid ratio, and so on, involved in the extraction process, on the yield of the extracting agent by comparing two factors at a time while rest of factors are set to zero (Liu et al. 2020). In the current experiment according to the regression equation, all 3D response surface figures were drawn. The 3D response surface plots clarified the influence of some experimental factors on the anthocyanins yield extracted from *B. kaschgarica* dried fruit powder, as well as the interaction between the two experimental factors was also marked highly. Blackhall et al. (2018) reported that increasing the solid‐to‐liquid ratio up to 1:10 results in an increased yield of anthocyanins however, a further increase in the ratio results in a significant decrease of the total anthocyanins contents of sweet cherry ( *Prunus avium* L.). Similar results were presented by Dumitraşcu et al. (2019), during the extraction of anthocyanins from cornelian cherry fruits. Our findings are parallel to the results of these previous studies.

Anthocyanins are plant secondary metabolites found in high concentrations in flowers and fruit of plants (Mattioli et al. 2020). Many studies conducted as in vitro, in vivo, and human trials explored different health beneficiary effects of anthocyanins, that is, their antioxidant activity, and antimicrobial activity, as well as been found an effective source against the encountering of different non‐communicable diseases such as metabolic diseases, cardiovascular diseases, and cancer (He and Giusti 2010). In the current study, we aimed to evaluate the antihypertensive effect of purified extract of *B. kaschgarica* anthocyanins. For this purpose, we used the SHR rat model. The SHR rats are basically inbreds of WKY and Wistar rats. In these rats without any pharmacological, physiological, or surgical intervention hypertension develops at the age of 4–6 weeks. The significance of this model has been attributed to the pathophysiology's resemblance to essential hypertension in humans (Dornas and Silva 2011). In the current experiment after treatment for 4 weeks, the highest dose of purified extract of *B. kaschgarica* anthocyanins was found the most effective dose against SBP which results were similar to the SHR + CAP group, while in DBP a significant decrease for the same dose was noticed from Week 6 in comparison to the SHR group. On the other hand, in hemodynamic studies, the BKRA‐H group was found effective against all the parameters (*p* < 0.01), while the BKRA‐M was noticed effective in reducing the values of SBP, LVSP, LV + dp/dtmax, and LV‐dp/dtmax (*p* < 0.01).

Cardiovascular diseases rank as the leading cause of death and the primary risk factor for cardiovascular diseases is high blood pressure (Kim et al. 2020). The rise in pulse pressure may result in heart attack or any other cardiovascular conditions (Oparil, Zaman, and Calhoun 2003). Blood pressure regulation involves a complex interplay of physiological mechanisms, encompassing various organs, hormones, and catecholamines (Drummond et al. 2019). It is known that the RAS is a biological and hemodynamic mechanism of regulating blood pressure (Bullock et al. 2001), in which the REN converts the angiotensinogen in the circulatory system into Ang‐I, which is further converted to Ang II by ACE that binds to angiotensin II type 1 receptor and induces vasoconstriction resulting in hypertension (Tipnis et al. 2000). While on the other side in the RAS, a vasodilating peptide like Ang‐(1–7) is produced that acts as a vasodilatory agent by binding to the angiotensin II type 2 receptor (Ferrario et al. 1997). In our study, we found that the serum level of the vasoconstriction peptides REN, ACE, and Ang‐II was significantly decreased in the SHR + CAP, SHR + BKRA‐M, and SHR + BKRA‐H groups (*p* < 0.001). At the same time, it was also noticed that the serum level of Ang‐(1–7) was significantly raised in the SHR + BKRA‐M and SHR + BKRA‐H groups in comparison to the SHR group (*p* < 0.001). Alternatively, vascular endothelial factors like ET‐1 and NO also play an important role in the regulation of blood pressure, higher level of ET‐1 and lower level of NO promote vasoconstriction as a result of which hypertension develops (Kostov 2021). Results of the current study show that serum concentration of the vascular contractile factor ET‐1 was significantly reduced in the SHR + CAP, SHR + BKRA‐M, and SHR + BKRA‐H groups (*p* < 0.01), while simultaneously the level of NO which is a vasodilatory factor was found significantly increased in the SHR + CAP, SHR + BKRA‐M, and SHR + BKRA‐H groups (*p* < 0.001). Our experiment results suggested that the purified extract of *B. kaschgarica* anthocyanins is an effective source against hypertension. However, further research is required to investigate and clarify the antihypertensive mechanism of the fruit extract.

## 5 Conclusion

In the current study, optimal experimental parameters of UAE were established for extraction of anthocyanins from *B. kaschgarica* fruit by employing RSM approaches, and the purified extract was evaluated for antihypertensive effect in SHR rats. It was clarified in the results that ethanol concentration, solid‐to‐liquid ratio, temperature, extraction time, and HCL volume significantly influence the extraction rate of anthocyanins. RSM was successfully employed for the optimization of the best parameters for the extraction and several factors were assessed. The results demonstrated that the highest contents of anthocyanins of *B. kaschgarica* can be obtained at 55% ethanol volume, 1:20 solid–liquid ratio, 55 min extraction time, and 0.7% HCL volume. Furthermore, the purified extract of *B. kaschgarica* anthocyanins was found highly effective in reducing SBP and DBP in the rats during 12 weeks of treatment. In addition, the reversal in the left ventricular function showed that the treatment was able to improve the left ventricular contractility. Moreover, the decrease in the plasma REN, ACE, Ang II, and ET‐1 while improvement in Ang‐(1–7) and NO indicates the role of the extract as an antihypertensive agent through interfering with the RAS and vascular endothelial factors. In conclusion, this study confirms the utilization of ultrasonic extraction technology to derive anthocyanins as a bioactive compound from *B. kaschgarica* aligning with industrial demand and sustainable development goals. The results of the current study suggest that *B. kaschgarica* fruit extract could be beneficial for improving cardiovascular health and hypertension management. Furthermore, it holds potential as a functional food ingredient for health conscious consumers. However, further investigation is required to clarify the mechanism of action of *B. kaschgarica* fruit extract in hypertension management.

## Author Contributions

**Nawaz Khan:** conceptualization (equal), data curation (equal), investigation (equal), methodology (equal), validation (equal), writing – original draft (equal), writing – review and editing (equal). **Maierdan Yusufu:** conceptualization (equal), formal analysis (equal), methodology (equal), software (equal). **Zeynab Ahmadova:** conceptualization (equal), methodology (equal), writing – original draft (equal), writing – review and editing (equal). **Nulibiya Maihemuti:** data curation (equal), resources (supporting), writing – review and editing (supporting). **Sendaer Hailati:** conceptualization (supporting), investigation (supporting), software (supporting), writing – original draft (supporting). **Ziruo Talihat:** formal analysis (supporting), methodology (supporting), validation (equal), writing – original draft (supporting). **Nuerbiye Nueraihemaiti:** data curation (equal), investigation (equal), validation (equal). **Dilihuma Dilimulati:** data curation (supporting), methodology (supporting), visualization (supporting). **Alhar Baishan:** formal analysis (supporting), investigation (supporting), software (supporting). **Li Duan:** conceptualization (equal), project administration (lead), resources (lead), validation (lead), writing – original draft (equal). **Wenting Zhou:** conceptualization (lead), funding acquisition (lead), project administration (lead), supervision (lead), writing – original draft (lead), writing – review and editing (lead).

## Ethics Statement

The animal study was reviewed and approved by the Animal Ethics Committee of Xinjiang Medical University.

## Conflicts of Interest

The authors declare no conflicts of interest.

## Data Availability

The study's supporting data are accessible upon request from the corresponding author.
